# Environment-Specific vs. General Knowledge and Their Role in Pro-environmental Behavior

**DOI:** 10.3389/fpsyg.2019.00718

**Published:** 2019-04-02

**Authors:** Sonja Maria Geiger, Mattis Geiger, Oliver Wilhelm

**Affiliations:** ^1^Institute for Vocational Training and Work Studies, Technische Universität Berlin, Berlin, Germany; ^2^Psychological Institute, University of Ulm, Ulm, Germany

**Keywords:** environmental knowledge, general knowledge, domain-specificity, environmentally significant behavior, structural equation modeling

## Abstract

Environmental knowledge has been established as a behavior-distal, but necessary antecedent of pro-environmental behavior. The magnitude of its effect is difficult to estimate due to methodological deficits and variability of measures proposed in the literature. This paper addresses these methodological issues with an updated, comprehensive and objective test of environmental knowledge spanning a broad variety of current environment related topics. In a multivariate study (*n* = 214), latent data modeling was employed to explore the internal factor structure of environmental knowledge, its relationship with general knowledge and explanatory power on pro-environmental behavior. We tested competing factor models and uncovered a general factor of environmental knowledge. The main novel finding of the study concerns its relationship with general knowledge. Employing an established test of general knowledge to measure crystallized intelligence revealed a near perfect relationship between environmental and general knowledge. This general knowledge (including the environmental domain) accounted for 7% of the variance in environmentally significant behavior. Age, additionally to acquired education, emerged as a common predictor for both general knowledge and environmentally significant behavior. We discuss the consequences of the strong relation between general and environmental knowledge and provide a possible explanation for the positive age-environmental conservation relationship reported in the literature.

## Introduction: the Role of Knowledge in Pro-Environmental Behavior

Knowledge about environmental issues is thought to be a precondition for meaningful pro-environmental behavior and its transmission is considered a key component and criterion for successful implementation of environmental education programs (UNESCO, 2005; Heimlich and Ardoin, 2008; Kaiser et al., 2008). A moderate, positive influence of domain-specific environmental knowledge has been reported in early research on pro-environmental behavior (Hines et al., 1987) and was replicated in various studies since (Kaiser and Frick, 2002; Frick et al., 2004; Meinhold and Malkus, 2005; Geiger et al., 2014, 2018). Other studies have found very small cross-sectional relationships (Roczen et al., 2013; Braun and Dierkes, 2017; Otto and Pensini, 2017) and the limited use of knowledge interventions on behavior change has been discussed since the seminal paper of Kollmuss and Agyeman (2002). Kaiser and Fuhrer (2003) propose that environmental knowledge, as a distal predictor of environmental behavior, is systematically underestimated and therefore not as prominently researched in environmental behavior studies as normative or attitudinal aspects. If environmental knowledge is studied, it is often restricted to subjective self-report measures of ability (e.g., Duerden and Witt, 2010; Milfont, 2012) or conflated with subjective evaluations of environmental issues as so-called problem awareness (e.g., Bamberg and Möser, 2007). Due to these methodological issues, it is impossible to derive sound conclusions about the influence of actual environmental knowledge on pro-environmental behavior. In cases where it is assessed objectively, different knowledge types have been suggested with insufficient testing of this claim (e.g., Roczen et al., 2013; Geiger et al., 2014; Liefländer et al., 2015) and the relationship to an overarching general knowledge factor has not been tested up to date, although general knowledge is a widely researched topic in psychological research as an individual differences with far-reaching practical implications. The main aim of this paper is to explore the relation of environmental knowledge to general knowledge and the predictive power that each has on environmentally significant behavior. Toward this end we present an updated, objective environmental knowledge test, and investigate its measurement properties that challenge the distinction of different knowledge types in existing models of environmental competence.

### Methodological Issues of Knowledge Assessment

Without a precise definition of knowledge, it is impossible to meaningfully investigate the structure of environmental knowledge and its predictive power on corresponding behavior. Knowledge is the result of a person’s lifelong learning process, i.e., the voluntarily accessible and organized accumulation of veridical information (facts, rules, etc.). Veridical means that the information is unequivocally true or false. To evaluate an individual’s performance in a knowledge test, her responses must be compared to the veridical answer of a given item or question, and consequently be rated as either “correct” or “wrong” (Cronbach, 1949). In contrast, many studies in environmental research use confidence or agreement ratings that assess self-concepts of one’s own knowledge, i.e., “I can explain what the term ecology means,” (Duerden and Witt, 2010), “How well-informed do you consider yourself to be on global warming and climate change?” (Milfont, 2012), or “How would you rate your knowledge/ability/awareness about composting/recycling/sustainability…?” (Redman and Redman, 2014). These tests do not measure actual knowledge, but a meta-representation of subjective knowledge (Metcalfe and Shimamura, 1996). Basic research on self-reports of cognitive abilities shows weak to zero relations with objective ability measures (Mabe and West, 1982; Jacobs and Roodenburg, 2014), corroborated recently for sustainability related knowledge (Effeney and Davis, 2013). These low correlations are suggestive of that the so-called Dunning-Kruger effect also exists in the environmental domain, where especially novices in a certain domain grossly overestimate their own expertise (Kruger and Dunning, 1999; Sanchez and Dunning, 2018).

Another prominent approach in environmental research is the subjective evaluation of the severity of a certain environmental issue on again, a Likert-type agreement rating scale, e.g., “Air pollution from private car use is a threat to plants and animals in the world” (e.g., Eriksson et al., 2006). This approach is problematic when it is conflated with objective knowledge measures, as in the meta-analysis undertaken by Bamberg and Möser (2007), for example. Their conclusion of an indirect influence on behavior via normative variables and feelings of guilt cannot be definitely attributed to objective, verifiable knowledge. Likewise, the meta-analysis’s results could also reflect effects of subjective feelings about whether an environmental issue is a problem. As with confidence ratings, a recent study by Ünal et al. (2017) could show that problem awareness correlated only weakly with general knowledge on climate change consequences and not at all with more specific knowledge on the topic.

These examples show that subjective measures of environmental “informedness” and problem awareness are not the same as objective knowledge. Despite recent evidence, that these two types of knowledge are subject two different psychological processes (Dunning and Helzer, 2014) and might contribute to behavior in different ways, there is not much systematic investigation into these differences in the environmental domain. One notable exception is a recent study by Passafaro and Livi (2017) that also evidenced an only moderate correlation between perceived and actual recycling skills (*r* = 0.33). More interestingly, they found that perceived skills are indicative of if people are likely to act at all (motivation), and actual skills relate to how well they will perform (accuracy).

To advance systematic research on the role and structure of environmental knowledge in according behavior, objective instruments are indispensable, even if their development is more complex, as they have to be validated for veridicality, content, uniqueness of correct answers, difficulty levels of adequate distractors (false answer options) or are based on highly ecologically valid simulation tasks (Passafaro et al., 2016). This study contributes to this endeavor with the actualization and advancement of such an instrument based on existing instruments (Kaiser and Frick, 2002; Geiger et al., 2014; Braun and Dierkes, 2017).

### Specific Types of Environmental Knowledge?

The environmental-competence model by Kaiser and colleagues (Frick et al., 2004; Kaiser et al., 2008; Roczen et al., 2013) is based on an objective knowledge test that advocates domain-specific knowledge in environmental education. It specifies three different knowledge types that have shown to affect environmental behavior to different degrees. According to this model, system related knowledge comprises declarative knowledge about the ecological system and natural laws (e.g., “What are constituents of biodiversity?”). Action-related, procedural knowledge about possible actions for environmental conservation (e.g., “Does buying organic food help to conserve biodiversity?”) is expected to exert a strong direct effect on behavior. Effectiveness knowledge relates to information about relative effects of different behaviors (e.g., “Is buying organic food more effective to conserve biodiversity than giving up certain products?”) and is assumed to have a motivational function on engaging in relevant behaviors.

The assumed structure of three facets of environmental knowledge – system, action, effectiveness – has rarely been empirically tested against a parsimonious one factor model, where all items are modeled onto a single dimension. When it has, only one study found marginally better fit parameters for the three factor model (Frick et al., 2004). One study found no difference (Kaiser and Frick, 2002) and further studies did not explicitly test different alternatives (Roczen et al., 2013; Geiger et al., 2014; Liefländer et al., 2015). Taken this sparse evidence, it remains unclear whether a multifaceted structure of environmental knowledge, although theoretically plausible, is an empirically warranted, appropriate model of people’s actual knowledge.

### General Versus Domain Specific (Environmental) Knowledge

Unlike research on environmental knowledge, investigating the structure and influence of general knowledge on behavioral outcomes has a long tradition in psychological research on individual differences (Cattell, 1971). In intelligence research, general knowledge is a well-established construct subsumed under crystallized intelligence (“gc”). It denominates the corpus of acquired factual knowledge and a person’s experience over a lifetime which predicts important outcomes in everyday life, such as academic achievement (Ones et al., 2005) or job performance (Hunter, 1986; Lievens and Patterson, 2011). While most cognitive abilities decline during adulthood, general knowledge grows with increasing age, up until late adulthood (Schroeders et al., 2015). In contrast, fluid intelligence (“gf”) comprises abilities such as reasoning, working memory capacity, etc., which are independent of a specific learning history (for a detailed description of both, see Carroll, 1993).

Theoretically, general knowledge might cover any domain, from knowledge about current events (e.g., celebrities) to detailed knowledge about decimals of π. The so-called “knowledge-is-power” hypothesis assumes that independent, domain-specific factors of knowledge are shaped by individual learning history in a certain domain, i.e., gaining expertise (Hambrick and Engle, 2002; Hambrick, 2003; Hambrick and Oswald, 2005) and that this expertise explains domain specific performance. However, factor analytic research puts the domain specificity into question (Hambrick and Meinz, 2011). In several large and broadly varying samples, a carefully developed gc test with multiple domains (e.g., history, medicine, literature, and many more distinct disciplines) always points to a general factor of gc with no evidence of domain specificity (Schroeders et al., 2013; Schipolowski et al., 2014a,b; Wilhelm et al., 2014).

Environmental knowledge might be considered another knowledge domain – a domain of specific interest in research concerning pro-environmental behavior or reaching sustainable development goals in a wider scope. Whether environmental knowledge is embedded in the general accumulated knowledge base of people has not yet been tested. The relationship between environmental and general knowledge is important for practitioners, as it will add empirical evidence to the discussion on appropriate approaches to environmental education. While an environmental-specific ability approach is advocated by Kaiser et al. (2008), recent literature on education for sustainable development advocates the concept of broad, content-independent competencies, such as perspective taking, participation, or handling of complex information (Haan et al., 2008; Michelsen and Fischer, 2017). If environmental knowledge shows systematic variation between participants after controlling for general knowledge, and if this residual variation predicts environmental behavior, the knowledge-is-power hypothesis will be supported. On the other hand, if environmental knowledge is empirically indistinguishable from general knowledge or does not account for environmental behavior and we will interpret this as evidence for a general knowledge model in explaining domain-specific behavior.

### The Predictive Power of Knowledge in Environmental Behavior

It has been convincingly shown that a simple information deficit model, where the provision of information is deemed sufficient to change the relevant behavior, is inappropriate (Kollmuss and Agyeman, 2002; Schultz, 2002). However, this evidence should not dismiss knowledge as a meaningful predictor of behavior altogether. Theoretically, knowledge can be considered a limiting factor for environmental behavior: if people do not know about environmental effects of their behavior, they cannot intentionally adjust their behavior toward generating less environmental impact. Kaiser (1998); Kaiser and Wilson (2000) was one of the first authors to present a comprehensive measure of ecological behavior, spanning behaviors from different areas of life as mobility, waste management, consumerism, energy consumption and vicarious behavior. Critique on their intention-based approach, where the intention to seek to minimize environmental damage solely defines pro-environmental behavior was currently raised (Steg and Vlek, 2009; Huddart Kennedy et al., 2013; Geiger et al., 2017). These authors call for inclusion of actual impact criteria in the investigation of environmental behaviors in the social sciences, such as e.g., the ecological or carbon footprint of the services and products consumed by people. The current paper applies such an impact-based idea, where environmental behaviors are selected for their actual high impact on the environment.

With regards to the role of knowledge for environmental behavior, two extensive meta-analyses on the explanation of pro-environmental behavior (Hines et al., 1987; Bamberg and Möser, 2007) have supported an indirect role mediated via moral norms, feelings of guilt, and/or intentions to act more environmentally friendly. Various empirical papers show that when knowledge is measured objectively, direct relationships can be observed, explaining anywhere between 3 and 24% of behavioral variance (Kaiser and Frick, 2002; Frick et al., 2004; Meinhold and Malkus, 2005; Roczen et al., 2013; Geiger et al., 2014, 2018; Braun and Dierkes, 2017). The notion of an indirect, necessary, yet insufficient role of knowledge to bring about informed behavior is also brought forward by Kaiser and Fuhrer (2003), along with three arguments on why the influence of knowledge on behavior has been systematically underestimated: (a) the existence of overruling more direct influences as situational restrictions, (b) inadequate assumptions about the structure of knowledge, and (c) the use of inadequate statistical procedures that do not account for measurement errors. In the current paper, we will scrutinize the latter two obstacles: we investigate the adequate knowledge structure using latent modeling techniques to see if an influence of knowledge on behavior is replicated under such circumstances.

## Research Goals

Responding to the methodological problems outlined in the introduction, the first goal of this study is to present an updated and expanded measurement instrument for objective assessment of environmental knowledge and to test its internal structure concerning content subdomains and knowledge types (cf. Kaiser et al., 2008). As an improvement compared to existing scales, which focus on natural science aspects of ecology, the new test includes current issues in sustainability related social, political, and economic topics (see Leach et al., 2013). As basic intelligence research has called into question the “knowledge-is-power” hypothesis, the second goal is to investigate whether environmental knowledge should be regarded an independent knowledge domain or a subdomain of general knowledge. Our third goal is to investigate whether environmental knowledge predicts pro-environmental behaviors under best of measurement conditions over and beyond the influence of general knowledge.

## Materials and Methods

### Procedure

An online survey in German language was conducted with the survey tool Unipark and published online from July to September 2015. The link was advertised to a larger German-speaking online community sample and additionally advertised on German social media webpages. The survey included a short sociodemographic questionnaire followed by three scales in the following order: general knowledge (Berliner Test zur Erfassung Kristalliner Intelligenz, BEFKI), the updated environmental knowledge test (EKT) and a short impact based scale of environmental behavior (SIBS). The study was conducted according the ethical guidelines for online studies of the German Society for Online Research (Deutsche Gesellschaft für Online-Forschung, 2007). Consent of each participant was requested in digital form on the first page of the survey and anonymity of participants was guaranteed. Participation was voluntary and 10 online vouchers worth 20€ were raffled among the participants who completed all measures and opted in for the raffle. Ethical approval was not required as per local legislation.

### Sample

The website of online study was accessed 443 times. The dataset was cleaned by excluding participants who dropped out before the end (*n* = 228, including instant drop-outs, not accepting study terms and conditions, study restarts, etc.), because study aborts must be treated as a reversal of participation consent. Furthermore, participants that responded to at least one out of four attention check questions erroneously (*n* = 1; an example item reads “This is an Attention Check, please respond with B”), or showed clear response patterns (*n* = 0, i.e., with little-to-no within person variance across response options) were excluded. Overall, 214 subjects remained and are included in all subsequent analyses. Mean age is 32.4 years (*SD* = 11.7 years, ranging from 18 to 92 years of age, German population median 18+: 50 years) and 131 participants are female. With respect to education (German population in parentheses, Statista, 2014), 5.9% (32.9%) have a basic school education, 16.8 % (29,4%) have a simple high school diploma, 39.2 % (29,5%) have passed high school with a university entrance diploma or comparable, and 38.3% possess a university degree. Our sample is thus younger and better educated than the German average.

### Measures

#### Environmental Knowledge Test (EKT)

The environmental knowledge test was constructed to cover relevant core areas of ecology, climate, resources, environmental contaminants/health, and consumption behaviors. Based on a wider understanding of sustainability explicitly endorsing a socio-economic dimension (Farley and Smith, 2013; Leach et al., 2013), we additionally included items measuring knowledge about sustainability-related societal and economic issues. The test was constructed in a multiple-choice format with one correct answer and three distractors (see Appendix A) to be comparable to the validated test of general knowledge in terms of form, language, and difficulty (see below and Appendix B).

In the first round, items from existing scales were screened for relevant content and compliance with test construction standards such as veridicality and comparability of distractors based on their wording. This step yielded 7 suitable items from the environmental knowledge scale by Kaiser and Frick (2002): items 1, 2, 3, 7, 13, 23, 32) 7 items from the EKLA scale (Geiger et al., 2014: items 6, 8, 15, 17, 18, 20, 21) and 3 items from Schahn’s scale (Schahn, 1999: items 24, 33, 34). Nineteen additional items were constructed on the basis of ecology and environmental study books, course curricula of German schools, and webpages of official environmental institutions (e.g., German federal environmental agency^1^), German federal ministry for the environmental, nature conservation, building and nuclear safety (BMU^2^).

The resulting 36 items can also be classified according to their knowledge type (cf. Frick et al., 2004), yielding 21 system knowledge items, 7 action-related knowledge items, and 8 effectiveness knowledge items.

Response format of all items was multiple choice and constructed or adapted to have one correct answer and three distractor answers. For a list of all German items included in the final form (*n* = 35, 1 item excluded and 3 more items adapted) with their according thematic clusters, see Appendix A in Supplementary Material. The English translation has not been validated and is only displayed for illustrative purposes.

#### General Knowledge (BEFKI gc)

One extensively validated instrument for the assessment of general knowledge is the *Berlin Test of Fluid and Crystallized Intelligence* (BEFKI gc, Schipolowski et al., 2013; Wilhelm et al., 2014). The crystallized intelligence section of this test comprises 64 items that capture general knowledge in a multiple choice form. The 16 content domains reflect the German national curriculum and comprise subjects from natural sciences (chemistry, physics, geography etc.) humanities (music, arts, history, etc.), and social sciences (finance, economics, religion, etc.). Confirmatory factor analysis (CFA) has repeatedly revealed a strong general factor with high reliability (ω = 0.88) and little domain specificity (Wilhelm et al., 2014). It is available in three versions for different education levels (until grade 8, grade 8 to 10, grade 11+), as well as in parallel versions for applied and research purposes. As we aimed for a wide age range (18+), and as gc increases until late adulthood, we used the version for grade 11+. For three example items of the domain music, history, and chemistry, see Appendix B in Supplementary Material.

#### Short Impact Based Pro-environmental Behavior Scale (SIBS)

The SIBS comprises five of the six behavioral areas mobility, private energy consumption, waste management, consumption choices, and social behaviors assessed by Kaiser (1998), plus nutrition, which was added because of the high environmental impact of nutrition-related behaviors alongside the consumption fields of housing and mobility (EEA, 2013; Thøgersen, 2014; Geiger et al., 2017). The items were selected according to their environmental impact in terms of greenhouse gas emission or ecological footprint. Within each domain, so-called “big points” (Bilharz and Schmitt, 2011) were addressed, as e.g., heating or solar energy production for private energy consumption, or airplane and individual motor travels for the mobility sector and meat consumption and regional food for nutrition (Lorek and Spangenberg, 2001; Tukker et al., 2010), with the exception of three general consumption and two social behaviors that do not have a quantifiable direct environmental impact (items 11–15). Response format was a 5-point Likert scale ranging from 0 = “never” to 4= “always.” Two dichotomous items on electricity-provision were combined into one 5-point scale -item (neither item affirmed = 0, renewable energy provider affirmed = 2, own solar panel = 3, both items affirmed = 4). Car use was assessed with three questions on ownership, gasoline usage and fuel type, that were combined to an overall 5-point index of car use. After excluding two items on food waste and use of deposit bottle due to negative loadings, the final form included 18 items (see Appendix C in Supplementary Material). With only two to four items per subdomain, it is a short scale for the assessment of environmentally relevant behaviors.

### Methodological Framework and Statistical Analysis

This study uses structural equation modeling as a framework, a modeling technique that allows to combine factor analytic with path analytic research questions (for an introduction see Kline, 2011). The factor analytic approach allows to test measurement models, i.e., if latent constructs (as e.g., environmental knowledge in our case) are measured appropriately with items that adequately represent the latent factor. The path or regressional analytic approach reveals underlying relationships between these latent factors in a structural model (e.g., how much behavioral variance is explained by knowledge). Model fit parameters are used to indicate the overall quality of a specified model including both aspects of the model, the measurement and structure model(s). Therefore, the measurement model of a latent variable, especially when using a new test, has to be tested separately before including it in an overall structural model, this was done here for environmental knowledge as well as for behavior.

The statistical analyses to this end were conducted using R 3.4.1 (R Core Team, 2014), setting the threshold of any statistical significance testing in this manuscript to α = 0.05. We calculated McDonald’s (1999) with the package semTools (version 0.4-13; semTools Contributors, 2016) as an adequate index of reliability for latent variable modeling (Sijtsma, 2009). Item analysis was conducted using the package psychometrics (version 2.2., Fletcher, 2010). To investigate the factor structure of the EKT, we tested nested confirmatory factor models using the DIFFTEST option in Mplus (Muthén and Muthén, 2010) due the dichotomous nature of our data (correct/wrong), requiring the use of Weighted Least Squares Mean and Variance estimation (WLSMV, Muthen et al., 1997). Other latent analyses were conducted using the package lavaan (Rosseel, 2012) in R with Maximum Likelihood (ML) estimation.

The CFA and structural equation models (SEM) in section “Relationship of Environmental Knowledge and General Knowledge” and “The Role of General Knowledge and Environmental Knowledge in Environmental Behavior” were conducted with parcels as indicators, as our sample was to small to estimate these models on an item level, which would have required 97 indicators (Little et al., 2002). Parcels as proportion of correct scores were built for all knowledge and behavioral items belonging to a content domain. For example, all knowledge items related to financial issues were combined to a finance parcel and all knowledge items related to climate change were combined to a climate parcel. We proceeded likewise with behavioral items. For thematic classification of environmental knowledge items, see Appendix A and for behavioral items, see Appendix C.

To evaluate the goodness of fit of our models, we use root mean square error approximation (RMSEA) and comparative fit index (CFI). RMSEA is an absolute index of model fit reflecting the discrepancy between observed and postulated model, and therefore should approximate 0 (“perfect fit”). The CFI, on the other hand, is an incremental index that compares the postulated model to a base model with uncorrelated factors and thus should approximate 1 for perfect fit. According to statistical conventions, a model reflects the correlational structure of the empiric data well (“good model fit”) if CFI ≥ 0.95 and RMSEA < 0.06 (Hu and Bentler, 1999) and reasonable well (“acceptable model fit”), if CFI ≥ 0.90 and RMSEA < 0.08 (Bentler, 1990; Steiger, 1990).

## Results

We will first present the results on the psychometric properties and descriptive results of the environmental knowledge test [see section “Psychometric Properties and Descriptive Results of the Test of Environmental Knowledge (EKT)”], followed by the results on the relationship between environmental knowledge and general knowledge (see section “Relationship of Environmental Knowledge and General Knowledge”), and end with the analysis on the predictive power of both on environmental behavior (see section “The Role of General Knowledge and Environmental Knowledge in Environmental Behavior”).

### Psychometric Properties and Descriptive Results of the Test of Environmental Knowledge (EKT)

We computed three nested confirmatory factor analyses to evaluate the factor structure of the newly developed EKT. Three items (numbers 14, 28, and 33) were excluded from all further reported analyses due to negative loadings in all tested models. On the remaining 33 items, we tested: (1) a g-factor model with all items loading on a single dimension; (2) a three factor model with knowledge type: system, action and effectiveness knowledge as factors (Frick et al., 2004; Kaiser et al., 2008; Roczen et al., 2013); and (3) a seven factor model with content domains as factors (c.f. Appendix A). In case of multiple factors, these were allowed to correlate. Results of the three models are summarized in Table 1.

**Table 1:** Model comparisons of competing factor structures of the EKT (item *n* = 33) ordered by declining parsimony.

^#^	Model type	*χ*^2^-Model test	RMSEA	CFI	χ^2^-Difference test to previous #
1	g-factor	*χ*^2^(495) = 562, *p* = 0.020	0.025	0.895	-
2	Three factors: knowledge types	*χ*^2^(492) = 558, *p* = 0.021	0.025	0.896	*χ*^2^(3) = 55.0, *p* = 0.174
3	Seven factors: content domains	*χ*^2^(474) = 539, *p* = 0.021	0.025	0.898	*χ*^2^(18) = 20.1, *p* = 0.325

*χ^2^-Difference tests were always conducted in contrast to the previous model. As the estimator was WLSMV, χ^*2*^-Difference tests were conducted using the MPLUS DIFFTEST option (Muthén and Muthén, 2010)*.

Fit indices, as well as the χ^2^-Difference Test, indicate that no model is clearly superior to any other model. The more complex models 2 and 3 revealed serious computation issues, namely a non-positive definite ψ matrix, Heywood cases, and correlations *r* > 1 between latent factors. In model 2, two of the three factor correlations were estimated larger than unity, whereas in model 3, 16 out of 21 factor correlations were larger than unity. The computational issues can be interpreted as indicators of over-factoring. Therefore, and for reasons of parsimony, we conclude that a g-factor solution is the appropriate representation of factor structure of the EKT. The final g-factor has acceptable reliability of ω = 0.737.

Considering overall model fit, RMSEA was good, but CFI was not, which might be due to five items (12, 18, 20, 22, 35) revealing low and non-significant loadings due to extreme item difficulties (very hard/very easy). However, our sample is not sufficiently representative (i.e., sample size, distribution of education, age) to conclude with certainty that these items should be removed as of yet. Therefore we suggest slightly modified versions of these items with easier i.e., harder distractors to be tested in future studies, as presented in Appendix A. Table 2 shows the descriptive results for the seven different content domains. Whereas our sample is very knowledgeable about issues on climate and environmental contamination, knowledge on resources and their limitations, as well as according consumption behaviors conserving them, are less prevalent.

**Table 2:** Descriptive results on knowledge in different content domains.

	Thematic domain	Mean	*SD*	Min	Max
1	Basic ecology	66.7%	16.8	20.0%	100%
2	Climate	82.0%	16.4	20.0%	100%
3	Resources	34.0%	25.0	0%	100%
4	Consumption behavior	59.6%	18.1	0%	100%
5	Society/politics	74.9%	24.4	0%	100%
6	Economy	80.1%	25.2	0%	100%
7	Environmental contamination	86.0%	19.4	0%	100%
	Environmental knowledge overall	68.3%	13.1	18.2%	97%

*The descriptive results are displayed for applied contexts, factor-analytic results show that they all belong to a general knowledge factor gc*.

The final EKT (without items 14, 28 and 33) showed no evidence of floor or ceiling effects. Mean item difficulty (i.e., solving probability) was at *M* = 0.686, with a standard deviation of *SD* = 0.131. Mean item discrimination (i.e., the factor loading, which is the biserial item-factor relation in the model with WLSMV estimation) was good with *M* = 0.443 and a standard deviation of *SD* = 0.217. Consequently, we consider the item discrimination of the test to be acceptable overall.

### Relationship of Environmental Knowledge and General Knowledge

Next, we evaluated the relation of our newly developed EKT with the established BEFKI gc measure of general knowledge, specifying one latent factor for each construct. Based on modification indices we accepted one error correlation between indicators of general knowledge (parcels finance and medical knowledge). The model had acceptable to good fit [*χ*^2^(228) = 348, *p* < 0.001; CFI = 0.926; RMSEA = 0.049]. The two latent factors were extremely highly correlated (*r* = 0.930).

As this correlation indicates very high redundancy of environmental knowledge to the broader theoretically underlying construct of general knowledge, we further investigated the specificity of environmental knowledge. Therefore, we estimated and compared two inferentially nested models: (1) a g-factor model with only one factor of general knowledge predicting all parcels and (2) a bifactor model with the g-factor as in model 1 and an additional orthogonal factor with loadings only from the EKT-Parcels. The results of this model comparison are presented in Table 3.

**Table 3:** Model comparisons of general knowledge structure (indicators are parcels; parcel *n* = 16+7), including vs. excluding environmental knowledge as specific factor.

^#^	Model type	*χ*^2^-Model test	RMSEA	CFI	*χ*^2^-Difference test to previous^#^
1	g-factor	*χ*^2^(229) = 355.4, *p* < 0.001	0.051	0.922	
2	Bifactor: general factor + nested environmental knowledge factor	*χ*^2^(222) = 339.4, *p* < 0.001	0.050	0.927	*χ*^2^(7) = 16.0, *p* = 0.025

*χ^2^-Difference test was conducted using the MPLUS DIFFTEST option (Muthén and Muthén, 2010)*.

Both models had very similar and acceptable to good fit. The ordinary χ^2^-difference test comparing both models was significant with *p* = 0.025. However, because the χ^2^-difference test has high power to detect negligible effects with larger sample sizes we also investigated the nested factor’s loadings and variance (Brannick, 1995; Kelloway, 1995). The mean factor loading was low with *M* = 0.210 and none of the loadings were significant. Consequently, the factor’s variance neither was significant, with *p* = 0.230 indicating that there is no specificity of environmental knowledge in general knowledge.

### The Role of General Knowledge and Environmental Knowledge in Environmental Behavior

Table 4 summarizes the descriptive results on all three test variables. The overall mean of correct answers was very high for general knowledge items (79.3%) and higher than that of environmental knowledge items (68.6%). Environmental behavior was on a moderate level, close to the verbal anchor “occasionally,” with highest prevalence for recycling and frugal behaviors and lowest prevalence for high cost behaviors such as owning a solar panel or donating money to environmental organizations. For the prevalence data of all 18 behaviors, see Appendix C.

**Table 4:** Zero order bivariate correlations and descriptive results for environmental and general knowledge and environmental behavior.

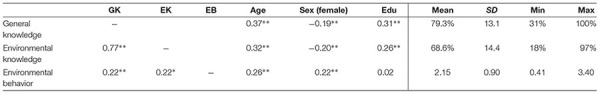

*For the knowledge scales, mean is presented in % correct answers, the behavior scale spans from 0 (“never”) to 4 (“always”), ^∗^*p* < 0.05, ^∗∗^*p* < 0.01, ^∗∗∗^*p* < 0.001*.

We initially planned to compare the prediction of general knowledge and environmental knowledge on environmentally significant behavior. Due to the above described findings of very strong relations between general knowledge and ecological knowledge and the non-significant variance of the environment specific knowledge, we take both constructs to be redundant; i.e., we assume that the EKT items are actually indicators of general knowledge, just as the BEFKI items are. Therefore, we modeled the EKT parcels as further indicators of a single general knowledge factor and studied the extent that such a global knowledge factor predicts environmentally significant behavior. To guarantee symmetry between measures, the SIBS scale was also modeled on the parcel level showing a good fit: *χ*^2^(9) = 15, *p* = 0.090; RMSEA = 0.057; CFI = 0.976.

**Figure 1:**
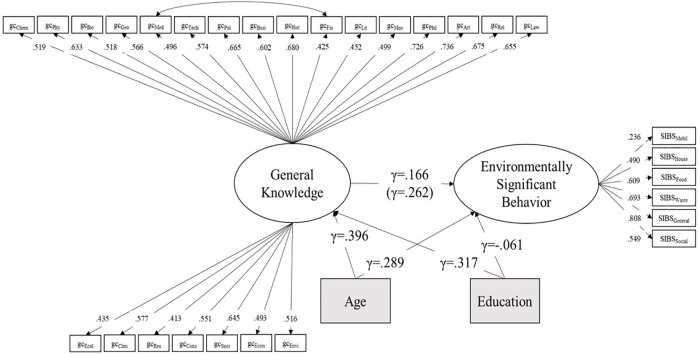
SEM model prediction of environmentally significant behavior through general knowledge and age as common cause. The *γ* of general knowledge predicting environmentally significant behavior in brackets is the value without including age and education. The value without brackets is the value when including age and education.

In the final SEM, shown in Figure 1, we tested a regression model with the general knowledge factor – now representing BEFKI and EKT parcels – predicting the latent sustainable behavior (SIBS) factor. The model had acceptable to good fit: *χ*^2^(375) = 537, *p* < 0.001; RMSEA = 0.045; CFI = 0.914. The standardized regression weight between sustainable behavior and general knowledge was small (γ = 0.261) but significant (*p* = 0.020).

In a last step, considering that our sample was not representative in age and education, we controlled the previous model for age and education. In a common cause model, we let age and education predict both sustainable behavior and general knowledge. The model fit was of mixed quality, with *χ*^2^(429) = 638, *p* < 0.001; RMSEA = 0.048; CFI = 0.896. Age and education were unrelated in our sample (*r* = 0.003, *p* = 0.964). Age had a significant positive effect on both, sustainable behavior (γ = 0.289, *p* = 0.017) and general knowledge (γ = 0.396, *p* < 0.001), where older participants showed more sustainable behavior and more general knowledge. Education had no effect on sustainable behavior (γ = -0.061, *p* = 0.445), but a significant positive effect on gc (γ = 0.317, *p* < 0.001). After controlling for age and education, the prediction of general knowledge on sustainable behavior dropped to γ = 0.166, which did not differ significantly from the weight obtained by the model without age and education [*χ*^2^(1) = 0.880; *p* = 0.348]. Additionally, entering gender into the structural model yielded a positive effect for female (γ = 0.608, *p* < 0.012) on standardized behavior.

## Discussion

Recent work has called for the construction of objective evaluation instruments to assess progress in sustainability education (Shephard et al., 2013; Décamps et al., 2017). We developed an environmental knowledge test from existing German tests to relate it to established measures of general knowledge. The test includes questions on a wide range of topics relevant to sustainability issues: basic concepts from ecology, climate, resources, consumption behavior, environmental pollution, economy, and society. The psychometric evaluation shows solid evidence for a unidimensional measure with acceptable reliability. Regarding the internal structure of environmental knowledge, neither different subdomains nor knowledge types according to Kaiser and colleagues (Kaiser and Frick, 2002; Frick et al., 2004) emerged as factors. This calls into question the usefulness of further distinguishing between knowledge forms in environmental education.

For our highly educated – and therefore somewhat variability restricted – sample, the mean probability of solving an item was 68.6%, allowing for discrimination concerning knowledge in less selected samples. The descriptive results hint on underexposed knowledge domains: whereas knowledge on climate issues and environmental deterioration is quite widespread in our educated sample, basic knowledge about issues on natural resources is least prevalent. Likewise knowledge on according consumption behaviors, i.e., action-related knowledge to preserve resources and protect the climate are not as widespread as desirable. We encourage use of the EKT for the evaluation of environmental education measures that aim at conveying environmentally significant knowledge in these different domains (see Appendix A for the full 35 item version, including seven improved items).

Our main theoretical finding, however, lies in the indistinguishability of environmental and general knowledge. The current data shows that in our age-heterogeneous sample, environmental knowledge is inseparable from general knowledge. With this, environmental knowledge is no different from other knowledge domains, such as medical or history knowledge, etc., and adds more evidence to refuting the “knowledge-is-power” hypothesis. Surprisingly, in broad samples, knowledge shows much less domain specificity than one would expect. The convergence of different academic knowledge domains to a general knowledge factor was shown in various studies (Schipolowski et al., 2014a; Schroeders et al., 2015), but is a novel finding in the area of environmental research.

General knowledge, including the environmental domain, predicted around 7% of variance in pro-environmental behavior. Using latent data modeling techniques, this (small) effect size is attenuated for measurement error and thus should be closer to a true value than simple correlation or regression coefficients based on manifest variables often used (for a critique see also Kaiser and Fuhrer, 2003). Albeit a small effect, this finding is noteworthy because it questions the claim that only behavioral-proximal knowledge (e.g., action knowledge) is relevant for corresponding behaviors. Our general knowledge test comprised very diverse and sustainability-unrelated subjects, ranging from humanities (e.g., history) to social sciences (e.g., religion). Providing evidence for the relationship between such a basic construct (general knowledge) and a very specific outcome (environmental conservation behavior) constitutes an example for a theoretically interesting and non-trivial finding, as opposed to correlational findings between similarly operationalized variables often presented in social science research (Fiedler, 2014).

On the other hand, despite the high level of general and environmental knowledge in our sample, pro-environmental behavior was merely average. The large amount of behavioral variance unaccounted for by knowledge backs up the notion that the influence of knowledge is partially overridden by potential intervening factors, such as normative influences (Bamberg and Möser, 2007), situational restrictions (Geiger et al., 2018), old behavior patterns (Klöckner, 2013) or simply net household income (Kleinhückelkotten et al., 2016). More recent work has called attention to the potential of environmental emotions, that could serve as a mediator to make environmental knowledge more relevant for actual environmental protection (Carmi et al., 2015; Otto and Pensini, 2017). Thus, according educational approaches seem more promising when taking into account a variety of variables. This stance is advocated by recent educational approaches on sustainable development (Michelsen and Fischer, 2017) that incorporate a wider range of general abilities to cope with environmental challenges. Educational aims beyond the transmission of knowledge comprise raising awareness, evolving empathic and social skills, and facilitating participation processes for sustainable solutions, for example. Such basic skills are typically acquired early in life and therefore taught in school and early education, as is general knowledge. Consequently, we recommend the implementation of interventions early on in school that facilitate sustainable behavior via basic abilities, such as general knowledge. As our study was cross-sectional, we cannot answer the questions whether conveying general abilities is more effective than domain-specific knowledge in environmental education, but our results do encourage further comparative research toward this end.

Age, independently of education level, emerged as a relevant predictor of both general knowledge and environmentally significant behavior, thus we might have inadvertently discovered an explanation for the positive age effect on environmental behavior: it is partially mediated by general knowledge that is accumulated over time (Horn, 2008). Although some older studies failed to detect a positive influence of age on environmental behavior (Hines et al., 1987; Ostman and Parker, 1987), other studies do (Gatersleben et al., 2002; Gilg et al., 2005), and a recent meta-analysis on the topic states “small but generalizable relationships […] that older individuals appear to be more likely to engage with nature, avoid environmental harm, and conserve raw materials and natural resources” (Wiernik et al., 2013, p. 826). Additional explanations for a direct age effect range from cohort effects rooted in the frugal upbringing of older generations (Olli et al., 2001; Wiernik et al., 2013), increased conscientiousness of older adults (Roberts et al., 2006), and/or an increased feeling of responsibility to leave an intact environment for future generations, the so called “legacy-motive” (Fox et al., 2010). Our findings suggest that the accumulation of knowledge over time (the age-dependent share of general knowledge) is a further possible explanation for the positive age-effect. Education, on the other hand, showed no direct effect on behavior. Accordingly, we take the level of education to be only indirectly relevant for environmental behavior in contributing to general knowledge of people.

A limitation of the present study is that it is confined to the current epoch and not set up to disentangle age- from cohort-effects. Over the last decades, environmental knowledge in Germany has become canonized, academic knowledge taught in schools and educational institutions. This reality makes our study highly cohort-dependent and studies from different decades or societies without a canonized environmental education might yield different results. A further limitation consists in the educated sample of the study. The convergence of knowledge domains might have been different in a less educated sample. Nevertheless, societal groups with high formal education and mediocre environmental behavior like our sample might be a relevant target group for environmental communication. A recent representative study from Germany showed, that overall energy consumption of households hinges strongly on formal education level and related net income (Kleinhückelkotten et al., 2016). Future research needs to clarify the independent influences of age, cohort, education and general knowledge on environmental behavior with a representative sample. As environmental knowledge, to a certain degree, depends on current developments and refers to regional aspects, according assessment instruments will have to be continuously updated and adapted. The EKT is an instrument that provides a valid item pool for such future work.

## Conclusion

In our study we found knowledge on a wide range of environmental topics to be a unidimensional factor inseparably linked to the general knowledge base of individuals. This finding challenges approaches that distinguish further between different types or content domains of environmental knowledge. When measured with an objective, reliable, and valid instrument, general knowledge accounts for a small portion of variance in environmentally significant behavior. The study adds evidence for refuting the “knowledge-is-power” hypothesis on the importance of domain-specific knowledge and supports an educational approach that goes beyond the transmission of environmental knowledge units aimed on specific environmental issues. The positive relationships between age, accumulated knowledge, and environmentally significant behavior yields an unexpected explanation for positive age effects in environmental conservation behavior.

## Author Contributions

SG performed the initial test construction assisted by MG and SG wrote the manuscript. MG performed the most data processing and statistical analyses and wrote part of the manuscript. OW guided data analyses and manuscript development.

## Conflict of Interest Statement

The authors declare that the research was conducted in the absence of any commercial or financial relationships that could be construed as a potential conflict of interest.

## Footnotes

1 www.umweltbundesamt.de

2 www.bmub.bund.de
