# Supplementary material for: Environment-Specific vs. General Knowledge and Their Role in Pro-environmental Behavior
Source: Front Psychol. 2019 Apr 2;10:718. doi: 10.3389/fpsyg.2019.00718 (PMC6454026; doi:10.3389/fpsyg.2019.00718)
Supplement: Supplementary file 1 [file Data_Sheet_1.pdf]

Appendix A – TEK Test of environmental knowledge and its content clusters

Content Domain	Item content (number)
Basic Ecology	Ground water (1), wind (2), soil formation (3) ecological niche (4), biodiversity (5)
Climate	Causes of global warming (6), carbon dioxide (7), renewable energy (8), consequences of global warming (9), mitigation of global warming (10)
Resources	Sealed areas (agriculture) (11), planetary boundaries (12), material recycling (13), agricultural water usage (14)
Consumption Behavior	Ecological footprint (15), eco labels (16), energy saving (17), transport efficiency (18), lighting efficiency (19), food packaging (20), energy usage (21), water usage (22), meat consumption impact (23)
Society /Politics	International organization (24), green politicians (25), Kyoto protocol (26), sustainability principle (27)
Economy	Sustainability reporting (28), externalized costs (29), green washing (30), corporate social responsibility (31)
Environmental contamination	Ground water hazard (32), hazardous waste (33), detergents (34), pacific garbage patch (35), castor transport (36)

Note: Items 14 and 33 were not included in the analysis due to negative loadings, here we present an adapted version with improved wording and better distractors. Items 12, 18, 20, 22 and 35 were only minimally adapted, introducing a different distractor to correct for extreme item difficulty. For future use we recommend this adapted version. Cultural-dependent items are marked with an asterisk.

1. Welche Aussage ist richtig?		1. Which statement is correct?	
☒	Grundwasser wird aus versickertem Oberflächenwasser (Regen, Flüsse, Seen) gebildet.	☒	Groundwater is composed of surface water that seeps into the ground (rain, rivers, lakes).
☐	Grundwasser kommt aus geologisch tiefen Schichten.	☐	Groundwater comes from deep geological layers
☐	Schadstoffe aus Fluss- und Regenwasser gelangen nicht ins Grundwasser.	☐	Pollutants from river and rain water do not get to the groundwater.
☐	Grundwasser ist alt und wird heute nicht mehr gebildet.	☐	Groundwater is old and is no longer being formed.

2. Wie entstehen Winde?		2. What causes wind?	
☐	Durch die Bewegung der Wolken.	☐	The movement of the clouds.
☒	Aufgrund von Temperatur- und Druckunterschieden in der Atmosphäre.	☒	Differences in temperature and air pressure in the atmosphere.
☐	Durch die Anziehungskraft des Mondes.	☐	The moon´s gravity.
☐	Aufgrund von Meeresströmungen.	☐	Ocean currents.

3. Wie lange dauert durchschnittlich die Bildung von 10 cm fruchtbarem Boden?		3. On average, how long does it take to form 10cm of fertile soil?	
☐	10 Jahre	☐	10 years
☐	80 Jahre	☐	80 years
☐	500 Jahre	☐	500 years
☒	2000 Jahre	☒	2000 years

4. Was ist eine ökologische Nische?		4. What is an ecological niche?	
☐	Der Ort, an dem eine Tierart die meiste Zeit des Jahres lebt.	☐	The place where an animal species lives the longest throughout the year.
☒	Das Zusammenspiel von belebter und unbelebter Natur an einem bestimmten Ort.	☒	The interaction of biotic and abiotic nature in a specific place.
☐	Der Ort, an den Tiere zurückkehren, um ihren Nachwuchs zur Welt zu bringen.	☐	The place where animals return to have their offspring.
☐	Der Ort, an dem sich Beutetierarten typischerweise verstecken.	☐	The place where prey species typically hide.

5. Welcher der folgenden Aspekte ist *nicht* Teil der biologischen Vielfalt?		5. Which of the following aspects is not a part of biodiversity?	
☒	Demographische Vielfalt	☒	Demographic diversity
☐	Artenvielfalt	☐	Diversity of species
☐	Ökosystemvielfalt	☐	Diversity of ecosystems

☐	Genetische Vielfalt	☐	Genetic diversity

6. Welches der folgenden Phänomene ist die Hauptursache für den Anstieg der Erdtemperatur in den letzten 20 Jahren?		6. Which of the following phenomena has been the main cause of global warming over the last 20 years?	
☐	Verminderung der Ozonschicht (das sog. „Ozonloch“).	☐	Reduction of the ozone layer (the so-called ozone hole).
☒	Vermehrter Ausstoß von Treib-hausgasen (der sog. „Treibhauseffekt“).	☒	Increased emissions of greenhouse gases (the so-called greenhouse effect).
☐	Veränderung der Meeresströmungen, z.B. „el Niño“.	☐	Changes in ocean currents, e.g. “el Niño”.
☐	Veränderung in der Kippung der Erdachse.	☐	Changes in the skewness of earth's axis.

7. Wofür steht die Abkürzung CO_2?		7. What is the meaning of the abbreviation CO_2?	
☐	Kohlenstoffmonoxid	☐	Carbon monoxide
☐	Treibhauseffekt	☐	Greenhouse effect
☒	Kohlenstoffdioxid	☒	Carbon dioxide
☐	Klimawandel	☐	Climate change

8. Welche Energieform zählt zu den erneuerbaren Energien?		8. Which energy form is a renewable form of energy?	
☐	Atomenergie	☐	Nuclear energy
☐	Erdöl	☐	Petroleum
☐	Erdgas	☐	Natural gas
☒	Erdwärme	☒	Geothermal energy

9. Welches Naturphänomen ist *nicht* auf die fortschreitende Erderwärmung zurückzuführen?		9. Which natural phenomenon is *not* attributed to global warming?	
☐	Gletscherschmelze	☐	Glacial melting

☐	Meeresspiegelanstieg	☐	Sea level rise
☐	Veränderung der Meeresströmungen	☐	Changes in ocean currents
☒	Erdbeben	☒	Earthquakes

10. Welche Handlung hilft *nicht*, den Treibhauseffekt zu reduzieren?		10. Which action does *not* help reduce the greenhouse effect?	
☐	Das Haus mit Solarthermie heizen.	☐	Heating a house with solar heat.
☒	Autos mit Katalysator fahren.	☒	Driving a car with a catalyzer.
☐	Im Alltag Strom sparen.	☐	Saving energy in everyday life.
☐	Bäume pflanzen.	☐	Planting trees.

11. Welches europäische Land verfügt über den höchsten Anteil versiegelter (=vom Menschen bebaute) Flächen an der Gesamtfläche des Landes?		11. Which European country has the most sealed areas (developed by humans) relative to its size?	
☐	Großbritannien	☐	United Kingdom
☐	Polen	☐	Poland
☒	Niederlande	☒	The Netherlands
☐	Italien	☐	Italy

12. Internationale Umweltexperten haben 9 Bereiche identifiziert, die entscheidend für die Stabilität des gesamten Erdsystems sind. In welchem Bereich wurde die Belastungsgrenze bereits überschritten?		12. International environmental experts have identified 9 factors that are crucial for the Earth system's stability. For which factor has the maximum load already been surpassed?	
☒	Klimawandel	☐	Climate change
☐	Süßwasserverbrauch	☐	Freshwater consumption
☐	Versauerung der Ozeane	☐	Ocean acidification
☐	Ozonabbau in der Stratosphäre (Ozonloch)	☐	Ozone depletion in the stratosphere (ozone hole)

13. Bei welchem Material wird durch Recycling gegenüber Neuherstellung am meisten Energie gespart?		13. For which material does recycling save the most energy in comparison to new production?	
☒	Aluminium	☒	Aluminum
☐	Glas	☐	Glass
☐	Weißblech	☐	Tinplate
☐	Papier	☐	Paper

14. Für die Produktion von welchem Lebensmittel wird am meisten Wasser benötigt? [1]		14. Which food requires the most water for production?	
☒	1kg Kaffee	☒	1 kg coffee
☐	1 kg Reis	☐	1 kg rice
☐	1kg Kartoffeln	☐	1 kg potatoes
☐	1 kg Äpfel	☐	1 kg apples

15. Was ist das ursprüngliche Prinzip der Nachhaltigkeit?		15. What was the original principle of sustainability?	
☐	Ressourcen müssen gerecht verteilt werden.	☐	Resources must be distributed fairly.
☐	Ressourcen müssen umweltfreundlich verbraucht werden.	☐	Resources must be used in an environmentally-friendly manner.
☒	Ressourcen dürfen nicht schneller verbraucht werden als sie nachwachsen können.	☒	Resources must not be used at a higher rate than they can reproduce naturally.
☐	Ressourcen müssen effizient eingesetzt werden.	☐	Resources have to be deployed efficiently.

16. Was versteht man unter dem CO_2-Fußabdruck eines Produktes?		16. What does the carbon footprint of a product mean?	
☐	Die typische Färbung des Himmels, die durch eine hohe CO_2 -Konzentrationen verursacht wird.	☐	The typical staining of the sky that is caused by high CO_2 concentrations.

general knowledge and environmental behavior

☒	Die Menge aller Treibhausgas-emissionen, die entlang des Lebenszyklus eines Produkts entstehen.	☒	The amount of all greenhouse gas emissions that is emitted over the lifecycle of a product.
☐	Die Menge von CO_2, die ein Produkt freisetzt, wenn es sich zersetzt.	☐	The amount of CO_2 a product emits when it corrodes.
☒	Die chemische Änderung, die durch CO_2 in der Atmosphäre hervorgerufen wird.	☐	The chemical change that is caused by CO_2 in the atmosphere.

17. Welches der folgenden Siegel steht für die höchsten Standards in der ökologischen Nahrungsmittelherstellung?*		17. Which of the following labels guarantees the highest standards for organic food products?*	
Bioland ☐	BIO nach EG-Öko-Verordnung ☐	☐	demeter ☒

18. Welche Handlung hilft *nicht*, im Alltag Energiekosten zu sparen? [1]		18. Which action does not help to save energy costs in everyday life? *	
☒	Wenn geheizt wird, Fenster dauernd gekippt lassen, als kurz ganz zu öffnen.	☒	Leaving the window tilted in the heating season instead of opening the window completely for a short time
☐	Gebäude gut isolieren, v.a. Dächer und Fenster.	☐	Insulating buildings properly, specifically roofs and windows
☐	Gefriergeräte eisfrei halten.	☐	Keeping freezers free of ice
☐	Die Waschmaschine mit eigenem Warmwasseranschuss versehen.	☐	Providing washing machines with a separate warm water supply

19. Welches Verkehrsmittel verursacht im Personennahverkehr die wenigsten Emissionen pro Personenkilometer?		19. Which type of transport produces the least amount of emissions per passenger and kilometer in short distance traffic?	
☒	Straßen, S- und U-Bahn	☒	Tram, subway, and urban train
☐	Auto	☐	Car
☐	Linienbus	☐	Public Transit Bus

☐	Motorrad	☐	Motorcycle

20. Welches Leuchtmittel ist am energie-effizientesten (am meisten Lumen/Watt)?		20. Which illuminant has the highest energy efficiency (the highest lumen/watt)?	
☐	Herkömmliche Glühlampe	☐	Conventional light bulb
☐	Halogenlampe	☐	Halogen lamp
☒	LED (Licht emittierende Dioden)	☒	LED (light emitting diode)
☐	Energiesparlampen	☐	Energy-saving light bulb

21. Welche der folgenden Getränke-packungen ist für die Umwelt am schädlichsten?		21. Which of the following beverage packaging is the most harmful to the environment?	
☐	Glasflasche, Mehrweg	☐	Reusable glass bottle
☐	Plastikflasche (PET), Mehrweg	☐	Reusable plastic bottle
☐	Getränkekarton (Tetrapak)	☐	Beverage carton (Tetrapak)
☒	Getränkedose	☒	Beverage can

22. Ein durchschnittlicher Haushalt in Deutschland verbraucht am meisten Energie für *		22. An average household in Germany consumes the most energy for…*	
☐	Beleuchtung	☐	Illumination
☐	Warmwasser	☐	Warm water
☒	Heizung	☒	Heating
☐	Elektrogeräte	☐	Electric devices

23. In welchem der folgenden Bereiche wird im Alltag am meisten Wasser verbraucht?*		23. In which of the following domains is the most water consumed in everyday life?*	
☒	Bei der Toilettenspülung.	☒	Flushing the toilet.
☐	Beim Essen und Trinken.	☐	Eating and drinking.
☐	Beim Geschirr spülen	☐	Washing dishes.

☐	Beim Wäsche waschen.	☐	Doing laundry.

24. Fleisch ist in der Produktion im Vergleich zur kalorienmäßig entsprechenden Menge Gemüse…		24. Considering producing the same amount of calories from vegetables, meat is …	
☐	Halb so umweltbelastend.	☐	Half as pollutive as vegetables.
☐	Gleich umweltbelastend.	☐	As pollutive as vegetables.
☐	Doppelt so umweltbelastend.	☐	Twice as pollutive as vegetables.
☒	Zehn mal umweltbelastender.	☒	Ten times more pollutive as vegetables.

25. Welche Organisation beschäftigt sich *nicht* vorrangig mit Umweltbelangen?		25. Which organization is not mainly concerned with environmental issues?	
☒	WHO (Weltgesundheitsorganisation)	☒	WHO (World Health Organisation)
☐	Greenpeace	☐	Greenpeace
☐	WWF (World Wildlife Fund)	☐	WWF (World Wildife Fund)
☐	Foei (Friends of the Earth International)	☐	Foei (Friends of the Earth International)

26. Wer wurde die/der 1. Grüne MinisterpräsidentIn Deutschlands?*		26. Who was the first Green Party Prime Minister of a German State?*	
☐	Bodo Ramelow	☐	Bodo Ramelow
☐	Renate Künast	☐	Renate Künast
☒	Winfried Kretschmann	☒	Winfried Kretschmann
☐	Joschka Fischer	☐	Joschka Fischer

27. Was ist das Kyoto-Protokoll?		27. What is the Kyoto Protocol?	
☐	Ein japanisches Gesetz zu Erneuerbaren Energien.	☐	A Japanese law on renewable energies.
☒	Die erste völkerrechtlich verbindliche Regelung der Treibhausgasemissionen in Industrieländern.	☒	The first compulsory regulation of greenhouse gas emission in industrialized countries.

☐	Ein internationales Protokoll über die Folgeschäden des Reaktorunglücks von Fukushima	☐	An international protocol concerning the consequential damages of the Fukushima reactor disaster.
☐	Das für Japan gültige, weltweit erste Protokoll zur Regelung des Emissionshandels.	☐	The first worldwide protocol for the regulation of emission trading valid for Japan.

28. Die Nachhaltigkeitsberichterstattung ist…	28. Sustainability reporting is…

29. Wie nennt man Kosten, die durch das wirtschaftliche Handeln eines Unternehmens entstehen, aber von der Allgemeinheit getragen werden?		29. What are costs due to economic activity of a company but paid by the public referred to as?	
☐	Internalisierte Produktkosten	☐	Internalized product costs
☒	Externalisierte Produktkosten	☒	Externalized product costs
☐	Ökologische Produktkosten	☐	Ecological product costs
☐	Unerlaubte Produktkosten	☐	Illegitimate product costs

30. Was ist mit dem Begriff „Greenwashing“ gemeint?		30. What does the term "Greenwashing" stand for?	
☐	Umweltfreundliche Waschgänge bei modernen Waschmaschinen.	☐	Environmentally-friendly washing cycles in modern laundry machines.
☒	Fälschlicherweise den Eindruck von Umweltfreundlichkeit zu erwecken.	☒	Falsely conveying the impression of being environmentally-friendly.
☐	Umweltfreundliche Produktions-vorgaben in der Textilindustrie.	☐	Environmentally-friendly production guidelines in the textile industry.
☐	Umweltfreundliche Entfernung von Graffitis.	☐	Environmentally-friendly removal of graffiti.

31. Was versteht man unter Sozialverant-wortung der Unternehmen (aus dem engl. "corporate social responsibility", CSR)?	31. Was does "corporate social responsibility" (CSR) stand for?

☒	Freiwillige Nachhaltigkeitsmaßnahmen von Unternehmen.	☒	Voluntary sustainability measures of companies.
☐	Betriebsinterne Sozialversicherungs-systeme von Unternehmen.	☐	Internal social security systems of companies.
☐	Ein Siegel für besonders soziale Unternehmen.	☐	A label for particularly social companies.
☐	Eine Unternehmenssteuer, die nur für soziale Zwecke verwendet werden darf.	☐	A business tax that can only be used for social purposes.

32. Wodurch wird das Grundwasser in der EU hauptsächlich belastet?*		32. What is the main pollutant of ground water in the EU?*	
☒	Durch das Düngen in der Landwirtschaft (z.B. Mit Nitraten).	☒	Agricultural fertilizers (e.g. With nitrates).
☐	Durch den zunehmenden Autoverkehr.	☐	Increasing car traffic.
☐	Durch die industrielle Luftverschmutzung.	☐	Industrial air pollution.
☐	Durch die Einleitung von Abwasser in die Gewässer.	☐	Discharge of wastewater into the water bodies.

33. Welcher Abfall muss als Sondermüll entsorgt werden?* [1]		33. Which item has to be disposed as hazardous waste?	
☐	Alte cds/ dvds	☐	Old cds/dvds
☐	Aufgeschäumte Styroporverpackung	☐	Foamed-up styrofoam packaging
☒	Durchgebrannte Energiesparlampen	☒	Used energy-saving light bulbs
☐	Leere Spraydosen	☐	Empty spray cans

34. Wasch – und Reinigungsmittel sollte man möglichst sparsam verwenden, weil...		34. Detergents and cleansers should be used as sparsely as possible, because …	
☐	starke Schaumbildung die Waschleistung vermindert.	☐	intense foaming reduces ~~the~~ washing performance.
☐	sie hohe Mengen an Schwermetallen enthalten.	☐	they contain high concentrations of heavy metals.
☐	die Abwasserrohre angegriffen werden.	☐	sewer pipes are affected.

☒	die Entfernung von diesen Stoffen in der Kläranlage schwierig ist.	☒	the extraction of these substances in purification plants is difficult.

35. In welchem Weltmeer befindet sich die größte zusammenhängende schwimmende Müllinsel ("the great ocean garbage patch")?[1]		35. The world's biggest continuous floating garbage patch ("the great ocean garbage patch") is located in which ocean?	
☒	Im Nordpazifik	☒	North Pacific
☐	Im Indischen Ozean	☐	Indian Ocean
☐	Im Nordatlantik	☐	North Atlantic
☐	Im antarktischen Ozean	☐	Southern Ocean

36. Wofür steht „Castortransport“?*		36. What does "Castor Transport" stand for?*	
☐	Für eine Versorgungslieferung an die Internationalen Weltraumstation ISS.	☐	A supply delivery to the International Space Station ISS.
☐	Für eine illegale Waffenlieferung.	☐	An illegal weapon delivery.
☐	Für Lebendtransporte von Schlachttieren.	☐	The transport of living slaughter cattle.
☒	Für den Transport von Behältern radioaktiver Abfälle.	☒	The transport of atomic waste containers.

*culture-specific items

Appendix B – Example items of the BEFKI scale

Music: Which composer is a famous representative of the “Vienna Classic”?	
☐	Johann Sebastian Bach
☒	Joseph Haydn
☐	Johannes Brahms
☐	Felix Mendelssohn-Bartholdy

History: After the “Battle of Leipzig”	
☒	Napoleon had to withdraw from Germany.
☐	The Allied Nation troops advanced to Berlin.
☐	Emperor Wilhelm II had to resign.
☐	Charlemagne was crowned emperor.

Chemistry: Salt crystals are made of	
☒	Ions.
☐	Molecules.
☐	Neutrons.
☐	Non-metal atoms.

general knowledge and environmental behavior

Appendix C – SIBS Scale

Five-point frequency scale (never- rarely-occasionally- often- always) prevalence as mean value

Nr.	Item	Mean prevalence	Content domain
1	For my regular travel to work or to shops, I use environmentally-friendly transport (bike, walking, or public transport).	2.76	Mobil
2	For my annual vacation, I fly abroad.*	2.11	Mobil
3	I buy organically-grown produce (e.g. Eco-labeled or from a health food shop).	2.37	Food
4	For my main meals, I eat meat.*	1.81	Food
5.	I eat regional fruits and vegetables when they are in season.	2.57	Food
6.	I keep the room temperature in my home so high in winter that I am warm without a sweater.	2.28	House
7.	I buy energy-efficient electro-domestics and light bulbs (A++ or A+ energy efficiency labels).	2.93	House
8.	I shower instead of taking baths to save water.	3.15	House
9.	I recycle materials (glass, paper, plastic packaging, metal).	3.30	Waste
10.	I avoid products with excessive packaging.	2.30	Waste
11.	I boycott shops that reportedly engage in environmentally-harmful business.	1.77	General
12.	I specifically buy products whose production and use impact the environmental as little as possible.	2.03	General
13.	I use things as long as possible instead of replacing them with a newer variant.	3.11	General
14.	I donate money to environmental organizations.	.74	Social
15.	I point out environmentally damaging behavior to people.	1.71	Social
16.	I own / use a car regularly (yes /no) if yes: this car uses**:<br>Diesel / E10 / regular /super / gas / electricity<br>Fuel consumption (6 categories according to type)	2.91	Mobil

Dichotomous items (yes/no), prevalence as item affirmed:

17.	I obtain electricity from a conventional / renewable energy provider.	26.6%	House
18.	My household has a photo-voltaic panel.	7.9%	

*inverse items, prevalence data for inversion: “I avoid…”.

** The high prevalence in this item is due to the student sample that to a large extent (50.5%) does not own a car
